# Precision medicine and novel molecular target therapies in acute myeloid leukemia: the background of hematologic malignancies (HM)-SCREEN-Japan 01

**DOI:** 10.1007/s10147-019-01467-1

**Published:** 2019-05-20

**Authors:** Kenichi Miyamoto, Yosuke Minami

**Affiliations:** Department of Hematology, National Cancer Center Hospital East, 6-5-1 Kashiwanoha, Kashiwa, 277-8577 Japan

**Keywords:** Leukemia, Precision medicine, Next-generation sequencing

## Abstract

The development of allogeneic hematopoietic-stem-cell transplantation has improved the prognosis of younger acute myeloid leukemia (AML) patients. However, the outcome of older AML patients remains poor. The majority of AML patients are elderly. For elderly AML patients unfit for intensive chemotherapy, less toxic single agent that targets a specific gene mutation or combination therapy with a single agent is needed. The role of chromosomal abnormalities and genetic mutations in leukemia has become more apparent, and detailed prognostic stratification based on the type of genetic mutation has been established. Next-generation sequencing (NGS) has been used for gene analysis of AML. In the future, the evaluation of biologically homogeneous population on the basis of chromosomal abnormalities and gene mutations will lead to a paradigm shift that will help in the development of optimized therapy. As rapid diagnosis of gene mutations is required by the clinical physicians to decide on induction therapy, it is important to have a swift turnaround time for comprehensive DNA sequencing to provide actionable data to clinical physicians. It is required to conduct a feasibility study to evaluate the turnaround time from sending the specimens to receiving the results while maintaining the quality of the specimens contributing to gene analysis. To detect infrequent gene mutations, investigators need to perform multicenter studies and/or cooperative-group trials with a certain sample size to examine the frequency of the gene mutations in elderly AML patients, enabling sufficient statistical power for meaningful comparisons.

## Introduction

Acute myeloid leukemia (AML) is a genetically heterogeneous malignant disorder of the haemopoietic stem cells. In Japan, the annual incidence of AML is 5.6/100,000 [1]. The annual incidence of AML has risen with the age of the population, reaching up to 10–17 out of 100,000 cases in patients who are more than 69 years old, whereas the annual incidence of AML is 0.6–6 out of 100,000 cases in less than 70 years of age of the generation. Therefore, majority of AML patients are more than 69 years old [1].

Although the standard treatment has not changed in the last 40 years [2], the development of allogeneic hematopoietic-stem-cell transplantation (allo-HSCT) and supportive care has improved the prognosis of younger AML patients ( < 60 years). However, the overall 5-year survival of older AML patients ( > 60 years) remains low (5–15%). Therefore, it can be said that effective standard treatment has not been established in elderly patients with AML [3, 4].

In recent years, the role of chromosomal abnormalities and genetic mutations in leukemia has become more apparent, and it is clear that these are associated with the treatment outcomes of leukemia. Next-generation sequencing (NGS) has been used for gene analysis of AML. At least more than one recurrent somatic mutation can be detected in almost all AML patients using comprehensive myeloid gene panels. Papaemmanuil et al. [5] identified one or more mutations in 96% of the patients using the myeloid panel comprising of 111 genes.

Moreover, numerous genetic mutations involved in epigenetic control mechanisms in AML have been identified by whole genome or exome sequencing. For example, genetic mutations have been seen in genes such as *TET2*, *IDH1/2*, or *DNMT3A* involved in methylation of DNA and histones, *ASXL1* or *EZH2* involved in chromatin modification, *STAG2* or *RAD21* involved in the formation of cohesin complexes during cell division, and *SF3B1* or *U2AF1* involved in RNA splicing [6, 7].

In 2013, whole exome sequencing of 150 AML patients and whole genome sequencing of 50 AML patients were carried out, respectively [8]. Mutations with an average of 13 amino acid substitutions per AML patient were observed in this report. Fewer mutations were involved in the development of AML compared to other forms of cancer. Furthermore, the frequency of occurrence of majority of the identified mutations was less than 5%. More than 10% of the gene mutations observed were found to be only three genes, namely *FLT3, NPM1, DNMT3A*.

On the other hand, by comprehensive genetic mutation analysis of a large number of AML cases, detailed prognostic stratification based on the type of genetic mutation has been established. Although the prognostic classification based on chromosome karyotyping has been used previously, an updated prognostic classification system has been proposed with the addition of chromosomal karyotyping in combination with assessment of the status of the gene mutation by European Leukemia Net (ELN), etc. In the newest classification of the ELN, more genetic mutations such as *RUNX1*, *ASXL1*, and *TP53* have been incorporated as prognostic factors [9].

It has been seen that the race of the patient influences the frequency of each gene mutation in AML. Comprehensive gene mutation analysis in Japanese AML patients has been performed using JALSG (Japan Adult Leukemia Study Group). JALSG conducted exhaustive gene mutation analysis by the target sequences for 55 genes using bone marrow samples of 197 AML patients who participated in AML201 study [10]. Although 44 genes were identified to be mutated using the target sequence for analysis, 5 gene mutations (*FLT3*, *NPM1*, *DNMT3A*, *CEBPA*, and *KIT*) were present at a frequency of more than 10% compared to the 3 genes found in the previous study [8].

Hence, the evaluation of biologically homogeneous population on the basis of race, chromosomal abnormalities and gene mutations will lead to a paradigm shift that will help in the development of optimized therapy for each of the homogeneous AML group.

## Significance of gene mutation analysis for AML

### Application to allo-HSCT

Gene mutational profiling before starting therapy for AML is becoming important as such profiling helps in deciding clinically whether to proceed with post-remission allo-HSCT therapy for the AML patients or not. Several studies have demonstrated cytogenetic abnormalities as prognostic factors and effective predictors in the setting of allo-HSCT in AML patients [11–14]. However, there are no studies evaluating the clinical benefits of allo-HSCT using comprehensive gene mutational data.

Recent reports show that the number of newer genetic abnormalities is growing. Schlenk et al. [15] conducted a study with 872 adults younger than 60 years of age with cytogenetically normal AML who were enrolled in AMLSG trials and demonstrated that in the patients with *NPM1* mutation without *FLT3* internal tandem duplication (ITD) mutations could not avail the clinical benefit of allo-HSCT (hazard ratio [HR] for the risk of relapse or the risk of death during complete remission 0.92, 95% CI 0.47–1.81).

Röllig et al. [16] analyzed the data of 304 patients with *NPM1* mutations with an intermediate-risk karyotype from the Study Alliance Leukemia AML 2003 trial and reported that the relapse-free survival (RFS) of *NPM1* mutant patients with an intermediate-risk karyotype was improved by allo-HSCT (HR 0.57, 95% CI 0.36–0.91, *P* = 0.017).

Extensive analyses were performed by Bornhauser et al. [17] using the data of 175 *FLT3*-ITD-positive patients with an intermediate-risk from the AML 96 study of the DSIL (German study initiative leukemia). In this analysis, the clinical benefit of allo-HSCT was shown in patients with *FLT3*-ITD mutations. The overall survival was not significantly different between *FLT3*-ITD-positive and -negative patients who have undergone allo-HSCT. In contrast, *FLT3*-ITD-positive patients receiving chemotherapy as consolidation therapy had a lower probability of survival (HR 2.2, 95% CI 1.4–3.5, *P* = 0.001).

Gröschel et al. [18] demonstrated that deregulated *EVI1* expression defines poor prognostic subsets among patients suffering from AML with t(11q23) and with t(9;11)(p22;q23). They divided 286 MLL-rearranged AMLs into three subgroups: t(9;11)(p22;q23) (44.8%), t(6;11)(q27;q23) (14.7%), and t(v;11q23) (40.5%)*.EVI1* expression was the prognostic factor for poorer overall survival (HR 2.06, 95% CI 1.28–3.31, *P* = 0.003), relapse-free survival (HR 2.28, 95% CI 1.35–3.86, *P* = 0.002), and event-free survival (HR 1.79, 95% CI 1.15–2.77, *P* = 0.009) within all t(11q23) AMLs. The outcome of *EVI1* positive patients with t(11q23) was significantly better after allo-HSCT in first complete remission (CR) versus other consolidation therapies (5-year OS, 54.7% vs 0%; *P* = 0.0006).

*RUNX1* mutations were analyzed in a group of 53 patients by Gaidzik VI et al. [19]*.* Out of 53 patients with *RUNX1* mutations, 32 attained a CR after induction chemotherapy (60.4%). In an exploratory analysis, they found *RUNX1* mutations were independent prognostic factors for shorter EFS in multivariable analyses for all patients with AML (APL excluded) (HR 1.494, *P* = 0.011). *RUNX1-*mutated patients who underwent allogeneic hematopoietic stem-cell transplantation had a better RFS in the subgroup analysis (the 4-year RFS, 52% vs 0%; *P* < 0.0001) [19]. Moreover, Chou et al. [20] retrospectively evaluated the clinical implications of 8 gene mutations including *RUNX1* mutation in 325 adult AML patients. They showed that the *RUNX1* mutation showed a favorable prognosis in the patients who received allo-HSCT (HR 0.33, 95% CI 0.120–0.932, *P* = 0.036) and poor prognosis in the patients who did not receive allo-HSCT (HR 1.74, 95% CI 1.02–2.67, *P* = 0.042) in multivariate analysis for overall survival.

Rucker et al. [21] examined patients with complex-karyotype AML and evaluated the effect of *TP53* alterations on the outcomes. In this study, *TP53* alterations were detected in 157 of 219 (72%) CK-AML patients. The overall survival of patients with *TP53* alterations was inferior compared with that of patients with wild type *TP53* in multivariate analysis (the 3 year-OS, 3% vs 28%; *P* < 0.0001). In addition, *TP53* alteration was also a poor prognosis factor in 30 CK-AML patients who received allo-HSCT in first CR (*P* = 0.04).

To examine the outcomes of the patients with *CEBPA* mutations, Schlenk et al*. *[22] analyzed the data from 4 Dutch–Belgian Haemato-Oncology Cooperative Group and Swiss Group for Clinical Cancer Research (HOVON/SAKK) trials and 3 AMLSG trials. Then, 124 patients who had AML with bi-allelic *CEBPA* mutations were included in this analysis. They reported that the RFS of the patients who underwent allo-HSCT was significantly superior to that of those who received chemotherapy. The 5 year-RFS of patients who underwent allo-HSCT was 73% (95% CI 54–86%) and the 5 year-RFS of those who received chemotherapy was 32% (95% CI 21–45%).

As described above, various gene mutations have been found as a prognostic factor and effective factor. However, the clinical outcome of AML patients with other mutations such as *ASXL1*, *DNMT3A*, *TET2*, *IDH1/2*, *WT1*, *EZH2*, and *PHF6* was unclear in the setting of allo-HSCT. Limited data are available to evaluate these mutations because the frequency of *ASXL1*, *TET2*, *IDH1/2*, *WT1*, *EZH2*, and *PHF6* mutations is not high. In addition, larger sample sizes are needed by the investigators to examine the clinical significance of a combination of these mutations.

To solve these problems, investigators need to conduct a comprehensive sequencing multicenter study which can collect more available biospecimens and clinical data.

### Application of novel agent

Gene mutational profiling can facilitate the introduction of new therapies that targets specific gene mutations in the relapsed/refractory AML patients. However, these agents also should be applicable for the treatment of newly diagnosed elderly patients who are unfit for intensive chemotherapy (in the setting of a clinical trial). There has been an increase in the number of targeted agents in AML.

Recently, some small-molecule FLT3 tyrosine kinase inhibitors (TKIs) such as midostaurin and quizartinib have been developed. Midostaurin inhibits a broad spectrum of kinases including FLT3, PDGFR, VEGFR2, KIT, and PRKC. In a total of 717 patients with *FLT3*-mutated AML, a combination of an oral multitargeted kinase inhibitor, midostaurin with cytotoxic chemotherapy (induction therapy with daunorubicin and cytarabine and consolidation therapy with high-dose cytarabine) resulted in better median OS (74.7 months vs 25.6 months, HR 0.78, *P* = 0.009) and event free survival [23]. Accordingly, midostaurin has recently been approved by the Food and Drug Association (FDA) and intensive chemotherapy with midostaurin is regarded as the standard treatment for adult patients undergoing first line treatment of *FLT3*-mutated AML. Quizartinib is an oral, highly potent and selective FLT3 inhibitor with clinical antileukaemic effectiveness. CRR was 46% (62/136) among 136 patients with *FLT3*-mutated AML in a phase 2 trial to evaluate the efficacy and safety of quizartinib in the relapsed or refractory setting [24]. Quizartinib has been also approved by FDA.

Isocitrate dehydrogenase (IDH) inhibitors have been also developed for both *IDH1* and *IDH2* mutations [25–27]. The inhibitor AG-120, which acts against mutated *IDH1*, showed efficacy [CR rate; 33%, median duration of response; 8.2 months (95% CI 5.6–12)] in AML [28]. Another IDH1 inhibitor, ivosidenib was promptly approval by the FDA for the treatment of patients with *IDH1*-mutated AML in the relapsed and refractory setting.

The promising activity of AG-221 (enasidenib) against mutated *IDH2* was shown in *IDH2*-mutated AML [25–27]. Preclinical data has demonstrated the efficacy of IDH2 inhibition in models of AML [29, 30]. AG-221 is a selective inhibitor of the mutant form of *IDH2*. The Overall response rate (ORR) and CR rate (CRR) for all relapsed/refractory AML patients was 40.3% and 19.3% in a phase 1/2 study to assess the maximum tolerated dose (MTD) and clinical activity of enasidenib in patients with mutant-*IDH2* relapsed/refractory myeloid malignancies [31]. In this study, a median OS of 9.3 months was observed in patients with relapsed/refractory AML, which can be considered as promising in this adverse patient subgroup. Consequently, the FDA approved enasidenib for patients with relapsed/refractory *IDH2* mutated AML.

IDH305 is a targeted inhibitor against mutated *IDH1R132.* The ORR of the relapsed and refractory AML was 33% (7/21) in a phase 1 study enrolling patients with myeloid malignancies, gliomas, and solid tumors [32].

At present, targeting agents are being typically developed and approved in the setting of relapsed or refractory AML. However, targeting agents are also in demand for untreated AML in older patients who are unfit for intensive chemotherapy. If a targetable mutation is detected in older AML patients, they should be considered for frontline treatment with a targeting agent that is less toxic than intensive chemotherapy in the context of a clinical trial.

### Necessity of an actionable mutation profiling multicenter study

The majority of AML patients are elderly. For elderly AML patients, intensive chemotherapy, which is standard treatment for younger AML patients, results in the addition of only a few months of prolonged lifespan as compared to palliative care. The number of phase III studies in which the subjects have been elderly patients in the last decade is less than 10 and the long-term survival rate of elderly AML patients has remained at 10–25%. It is hard to say if the standard treatment is effective in elderly patients with AML. Therefore, there is an unfulfilled medical need for an effective treatment for the elderly AML patients. In the future, for elderly AML patients unfit for intensive chemotherapy, less toxic single agent that targets a specific gene mutation or combination therapy with a single agent might become the standard therapy.

As described above, gene mutations can influence the prognosis of patients with newly diagnosed AML. As rapid diagnosis of gene mutations is required by the clinical physicians to decide on induction therapy, it is important to have a swift turnaround time for comprehensive DNA sequencing to provide actionable data to clinical physicians. It is required to conduct a feasibility study to evaluate the turnaround time from sending the specimens to receiving the results while maintaining the quality of the specimens contributing to gene analysis, especially when a physician would like to conduct a comprehensive genomic profiling overseas.

Many studies on the genetic abnormalities of AML have been reported from Western countries. However, the impact on the mutation frequency of each gene in AML differs with race. Although JALSG in Japan have reported genetic abnormality of AML, the subjects for this study were limited to less than 65 years of age [10]. Gene mutations associated with poor prognosis occurs at a high frequency in the elderly AML patients compared with the young patients [33]. In Japan, there is no report that has examined the frequency of these genetic abnormalities in AML including patients of 65 years of age or above.

Given the scarcity of certain gene mutations in AML, enrollment of patients for trials is often difficult. To detect infrequent gene mutations, investigators need to perform multicenter studies and/or cooperative-group trials with a certain sample size to examine the frequency of the gene mutations in elderly AML patients, enabling sufficient statistical power for meaningful comparisons.

### The outline of HM-SCREEN-Japan 01

(Hematologic malignancies) HM-SCREEN-Japan is a genetic screening project to establish precision medicine for AML patients conducted by National Cancer Center Hospital in collaboration with other institutions. The purpose of this project is to develop novel effective drugs against AML and to facilitate rapid clinical introduction of new diagnostic techniques such as multiplex somatic mutation diagnostic agents. HM-SCREEN-Japan 01 is an actionable mutation profiling multicenter study of patients with newly diagnosed acute myeloid leukemia unfit for the first standard treatment or relapse/refractory acute myeloid leukemia supported by Chugai Pharmaceutical Co., Ltd.

The objective of this study is to evaluate the frequency or the characteristics of cancer-related genome alterations in acute myeloid leukemia using a comprehensive genome profiling assay (FoundationOne®Heme, F1H).

The subjects enrolled for the HM-SCREEN-Japan 01 fulfilled the following criteria:

1. Histologically diagnosed with AML with bone marrow aspiration.
2. Either of the following was fulfilled.
  ① Patients with newly diagnosed AML unfit for standard treatment.
  ② Patients with relapse/refractory AML.
3. The specimens provided are fit for genetic analysis or not.
4. Obtained written informed consent.

The primary outcome is to estimate the frequency of each leukemia genome alteration. The secondary outcome is to evaluate the association between each leukemia genome alterations and clinicopathological characteristics or prognosis and quality of the specimens contributing to gene analysis. This trial has been registered at the UMIN Clinical Trials Registry as UMIN UMIN000035233 (https://www.umin.ac.jp/ctr/index.htm).

### Comprehensive genome profiling assay: FoundationOne®Heme

F1H was developed by Foundation Medicine Inc. (FMI) and provides a comprehensive genomic profile that applies next-generation sequencing with hybrid capture-based target enrichment approach to identify somatic genomic alterations in genes known to be unambiguous drivers of hematologic malignancies (leukemias, lymphomas, and myelomas) as well as sarcomas using formalin-fixed, paraffin-embedded specimens. Each profile simultaneously sequences the complete coding region of 406 genes as well as selected introns of 31 genes involved in rearrangements. F1H also investigates the RNA sequences of 265 commonly rearranged genes to better identify gene fusions (including de-novo and rare gene fusions). In addition to detecting rearrangements, F1H detects all classes of genomic alterations, including base substitutions, insertion and deletions, and copy number alterations using a small, routine clinical sample. All F1H samples were simultaneously profiled for tumor mutation burden (TMB) status. TMB is determined by measuring the number of somatic mutations occurring in the sequenced genes on the F1H profile and extrapolating to the genome as a whole.

## Conclusions

Evaluation of F1H for use in HM-SCREEN-Japan 01 enables analysis of cancer-associated genes which can be used as therapeutic targets and fusion genes which have been rarely identified so far in AML. The data on these genetic abnormalities are important epidemiological information for the development of novel therapeutics. It is also expected to lead to efficient patient enrollment in the early phase studies.
